# Pericardial effusion following percutaneous left atrial appendage closure using the LAmbre device

**DOI:** 10.3389/fcvm.2023.1188322

**Published:** 2023-06-06

**Authors:** Yibo Yu, Huimin Chu, Binhao Wang, Bin He, Guohua Fu

**Affiliations:** ^1^ Arrhythmia Center, Ningbo First Hospital, The First Affiliated Hospital of Ningbo University, Ningbo, China; ^2^ Key Laboratory of Precision Medicine for Atherosclerotic Diseases of Zhejiang Province, Ningbo First Hospital, The First Affiliated Hospital of Ningbo University, Ningbo, China

**Keywords:** pericardial effusion, predictor, LAmbre, left atrial appendage closure, atrial fibrillation

## Abstract

**Background:**

Pericardial effusion (PE) is an uncommon but serious complication that occurs following percutaneous left atrial appendage closure (LAAC). There are few data regarding PE following implantation of the LAmbre device for LAAC.

**Methods:**

Patients with nonvalvular atrial fibrillation (AF) undergoing percutaneous LAAC using the LAmbre device at the Arrhythmia Center of Ningbo First Hospital from October 2017 to March 2021 were retrospectively reviewed (*n *= 133). PE was defined as acute if diagnosed ≤7 days post LAAC (*n *= 3, 2.3%) or delayed if diagnosed >7 days post LAAC (*n *= 3, 2.3%). The clinical characteristics and procedural data were compared between patients with PE (PE group, *n *= 6) and without PE (non-PE group, *n *= 127). The predictors of PE were analyzed by logistic regression.

**Results:**

All patients with PE recovered following treatment by pericardiocentesis. Patients with PE were found to have a higher incidence of congestive heart failure (50.0% vs. 13.4%, *P *= 0.044) and had larger measured LAA orifice diameters (33.5 mm ± 6.0 mm vs. 28.3 mm ± 5.2 mm, *P *= 0.018) and landing zone diameters (27.8 mm ± 4.8 mm vs. 23.9 mm ± 4.8 mm, *P *= 0.054) compared with those without PE. The diameters of the device umbrellas (31.7 mm ± 5.6 mm vs. 26.9 mm ± 5.0 mm, *P *= 0.026) and covers (36.3 mm ± 4.6 mm vs. 33.4 mm ± 4.0 mm, *P *= 0.075) implanted were larger in the PE group compared to the non-PE group. Univariate logistic regression revealed that congestive heart failure (OR = 6.47, 95% CI* *= 1.21–34.71, *P *= 0.029) and LAA maximal orifice diameter (OR = 1.22, 95% CI* *= 1.02–1.45, *P *= 0.027) were both associated with PE following LAmbre device implantation.

**Conclusions:**

In this single-center experience, both acute and delayed PE were uncommon in patients with AF following LAmbre device implantation. Congestive heart failure and a larger LAA orifice were identified as predictors for the occurrence of PE.

## 1. Introduction

Percutaneous left atrial appendage closure (LAAC) is now considered a viable alternative to oral anticoagulation (OAC) for stroke prevention in patients with nonvalvular atrial fibrillation (AF) (1). Prior randomized controlled trials have demonstrated the safety and efficacy of LAAC (2, 3). Pericardial effusion (PE) requiring intervention is one of the most serious complications following LAAC. The reported rates of acute PE (≤7 days) in previous investigations range between 1.8% and 5% (4). Data regarding delayed PE (>7 days) following LAAC are limited. The incidence of delayed PE was reported as 0.8% in our recent article (5). In the Amulet IDE trial, the incidence of postprocedural PE was reported to be higher for procedures in which the Amplatzer Amulet occluder was implanted than when the Watchman device was used (6). Several factors associated with PE after LAAC have been reported, including older age, female sex, left ventricular function, paroxysmal AF, and higher CHA_2_DS_2_-VASc score (7, 8).

The majority of studies of PE were performed in patients with LAAC using either the Watchman or Amplatzer devices (8–11). The LAmbre device is a self-expanding, nitinol-based device consisting of an umbrella and a cover connected by a short central waist. It has been used in clinical practice since 2017 with a high success rate (12, 13). Previous investigations have shown that both acute and delayed PE can occur following LAAC with the LAmbre device (5, 12, 14). We retrospectively investigated the incidence and predictors of PE following the LAAC procedure with the LAmbre device in our institution.

## 2. Materials and methods

### 2.1. Study population

Patients with nonvalvular AF who underwent percutaneous LAAC using the LAmbre device at the Arrhythmia Center of Ningbo First Hospital from October 2017 to March 2021 were retrospectively studied. Periprocedural baseline data were collected to calculate the CHA_2_DS_2_-VASc score and HAS-BLED score. Preprocedural transthoracic echocardiography (TTE) was performed 24 h prior to the procedure to measure the left atrial (LA) diameter and the left ventricular ejection fraction (LVEF). The left atrial appendage (LAA) was visualized at 0°, 45°, 90°, and 135°, and the LAA orifice diameter and landing zone diameter were measured and recorded. The inclusion criteria for LAAC were as follows: age >18 years, CHA_2_DS_2_-VASc score ≥2 for males or ≥3 for females, presence of a contraindication to long-term OAC therapy, history of bleeding events while on OAC therapy, history of stroke while on long-term OAC therapy, and intolerance or refusal to take OAC. The exclusion criteria included presence of a thrombus in the LAA or LA detected by TEE, AF in the setting of moderate-to-severe mitral stenosis and/or in the presence of a mechanical heart valve, acute myocardial infarction or unstable angina, prior stroke or transient ischemic attack (TIA) within 30 days, and uncontrolled hemorrhagic disease. This study was conducted in compliance with the guidelines of the Helsinki Declaration. The study was approved by the Ethics Committee of Ningbo First Hospital. Written informed consent for the percutaneous LAAC procedure was obtained from all patients.

### 2.2. Percutaneous LAAC with the LAmbre device

All procedures were performed under local anesthesia guided by TEE or intracardiac echocardiography (ICE). The technique used for LAmbre device implantation have been published previously (12). To briefly summarize, intravenous heparin was administered to patients immediately after transseptal puncture aiming to achieve an activated clotting time of at least 250 s. The delivery sheath was then placed in the proximal part of the LAA. The device size was selected by the operator according to the measurements taken during intraprocedural echocardiography or LAA angiography. The distal umbrella was released into the LAA by pushing the device stepwise out of the delivery sheath. Subsequently, the sheath was withdrawn to expose the proximal cover, allowing it to expand in the LA and seal the LAA ostium. Echocardiography was performed to confirm satisfactory positioning of the device. A satisfactory device position was defined as absence of, or minimal contrast peridevice leakage (PDL) ≤3 mm into the LAA. A gentle tug test was performed to ensure device stability. Recapture or resizing of the device was attempted unless satisfactory device position was achieved. The C/O ratio was calculated as the diameter of the cover divided by the LAA maximal orifice diameter. The U/l ratio was calculated as the diameter of the umbrella divided by the maximum diameter of the LAA landing zone.

### 2.3. Follow-up

TTE was performed on the day following the procedure to detect the presence of PE. OAC was prescribed for 45 days after the procedure, followed by both aspirin and clopidogrel for 6 months, and subsequently aspirin or clopidogrel for the duration of the patient's lifetime. Repeat TEE was performed at 45 days, 6 months, and 12 months following the index procedure in order to evaluate the position of the device, PDL, and device-related thrombus (DRT). We considered major adverse events to include death, stroke/TIA, systemic embolism, device embolization, and major bleeding events. Major bleeding was defined according to the Bleeding Academic Research Consortium (BARC) criteria (type 3 or higher) (15).

### 2.4. Management of PE

Acute and delayed PE were defined according to the time of diagnosis following LAmbre device implantation (acute was defined as ≤7 days, and delayed defined as >7 days). Treatment of PE was dependent to the patient's symptoms, vital signs, and test results, as well as their physicians' judgment (5). If there were no signs of cardiac tamponade, intensive monitoring alone was performed. If cardiac tamponade was present, emergency pericardiocentesis was performed. If the vital signs were still unstable after pericardiocentesis, urgent cardiac surgery was recommended. Repeated TTE was also performed 1 or 2 weeks following discharge. The routine postimplantation follow-up strategy was then followed if the patient remained stable.

### 2.5. Statistical analysis

The study sample was divided into PE group and non-PE group based on the occurrence of PE. Normally distributed continuous variables are expressed as the mean (standard deviation), while the median (interquartile range) is used for variables with a skewed distribution. Categorical variables are expressed as absolute numbers (percentages). Continuous variables were compared using the *t-*test and Mann–Whitney *U*-test for normally and nonnormally distributed data, respectively. Categorical variables were compared using the Fisher's exact test. The predictors of PE were evaluated by logistic regression. According to a rule of thumb, logistic models should be used with a minimum of 10 events per predictor (16). Therefore, univariate logistic model would be run for the predictor of PE if the number of cases was not enough for the multivariate adjusted model. All analyses were performed by SPSS 19.0 (IBM, Armonk, NY, USA), and *P* < 0.05 was considered statistically significant.

## 3. Results

### 3.1. Baseline characteristics

**Figure 1:**
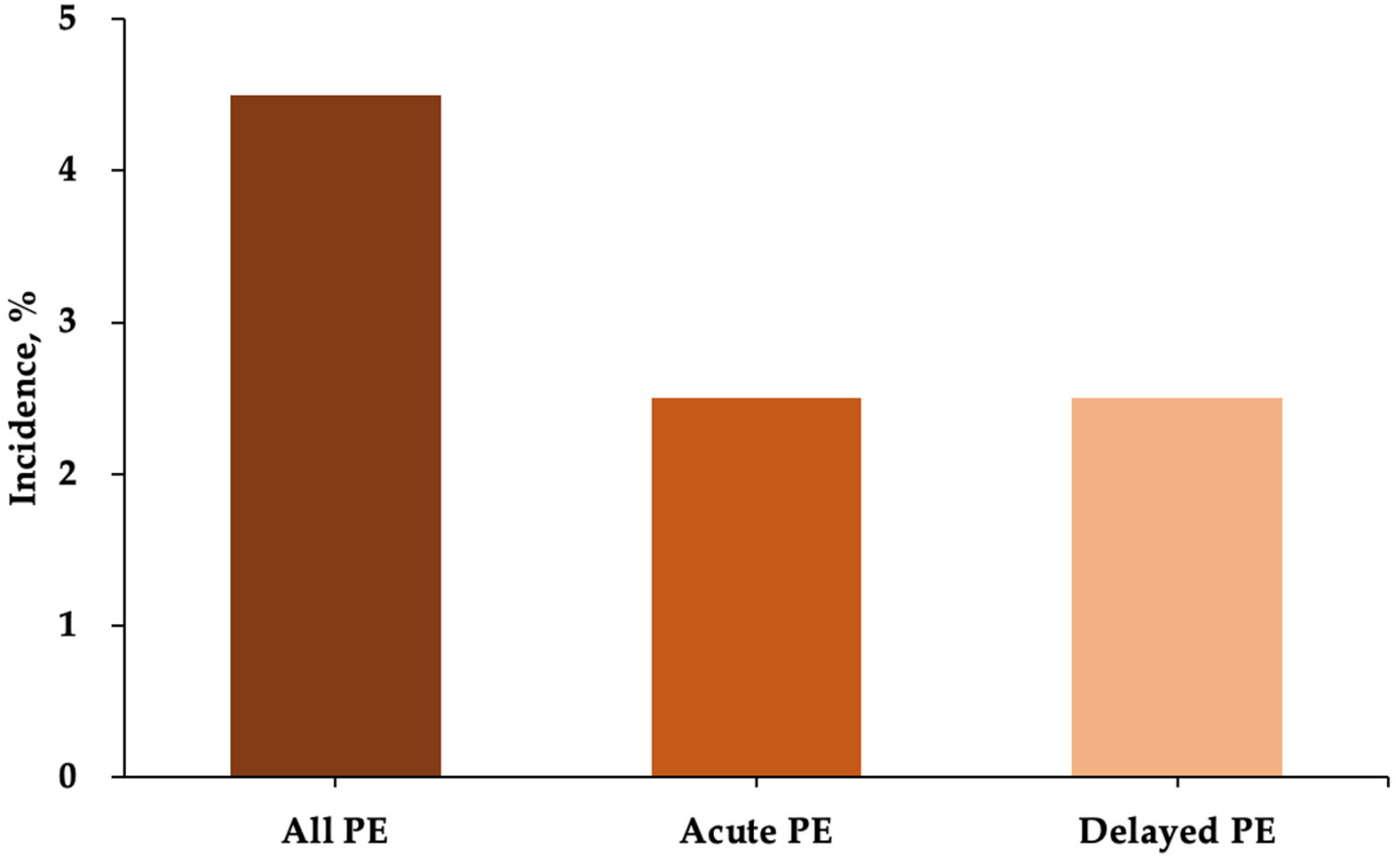
Incidence of PE after LAmbre device implantation. PE, pericardial effusion.

A total of 133 patients who underwent LAmbre device implantation were included in our analysis. Acute and delayed PE occurred in 3 patients each, resulting in a total incidence of 4.5% for all PE events (Figure 1). Baseline characteristics of the patients are shown in Table 1. Patients with PE had a higher percentage of congestive heart failure (50.0% vs. 13.4%, *P *= 0.044). The LAA orifice diameter (33.5 mm ± 6.0 mm vs. 28.3 mm ± 5.2 mm, *P *= 0.018) and landing zone diameter measured by TEE (27.8 mm ± 4.8 mm vs. 23.9 mm ± 4.8 mm, *P *= 0.054) were both greater in the PE group. The CHA_2_DS_2_-VASc score and HAS-BLED score were similar between the two groups.

**Table 1:** Baseline characteristics of the patients.

Variables	Non-PE	PE	*P* value
*n*	127	6	
Age, years	70.5 ± 8.6	71.7 ± 8.2	0.742
Female, *n* (%)	47 (37.0)	1 (16.7)	0.418
BMI, kg/m^2^	23.4 ± 3.2	23.5 ± 4.3	0.944
Paroxysmal AF, *n* (%)	21 (16.5)	1 (16.7)	1.000
Congestive heart failure, *n* (%)	17 (13.4)	3 (50.0)	0.044
Hypertension, *n* (%)	81 (63.8)	5 (83.3)	0.423
Diabetes mellitus, *n* (%)	26 (20.5)	1 (16.7)	1.000
Peripheral vascular disease, *n* (%)	90 (70.9)	4 (66.7)	1.000
Renal dysfunction, *n* (%)	25 (19.7)	2 (33.3)	0.601
Hematological disorder, *n* (%)	0	0	1.000
Anemia, *n* (%)	4 (3.1)	0	1.000
CHA_2_DS_2_-VASc score, points	4.7 ± 1.6	5.0 ± 0.6	0.292
HAS-BLED score, points	2.9 ± 1.0	3.2 ± 0.8	0.553
LA diameter, mm	45.7 ± 6.9	46.8 ± 11.0	0.714
LAA orifice diameter, mm	28.3 ± 5.2	33.5 ± 6.0	0.018
LAA landing zone diameter, mm	23.9 ± 4.8	27.8 ± 4.8	0.054
LVEF, %	60.8 ± 6.9	55.8 ± 9.0	0.091
Indications for LAAC*, *n* (%)			
Stroke/TIA on OAC	92 (72.4)	4 (66.7)	0.670
Bleeding on OAC	29 (22.8)	1 (16.7)	1.000
Intolerance to OAC	24 (18.9)	1 (16.7)	1.000
Refusal to OAC	12 (9.4)	1 (16.7)	0.467

AF, atrial fibrillation; BMI, body mass index; LA, left atrium; LAA, left atrial appendage; LAAC, left atrial appendage closure; LVEF, left ventricular ejection fraction; OAC, oral anticoagulation; PE, pericardial effusion; TIA, transient ischemic attack.

*For some patients >1 indication was reported.

### 3.2. Periprocedural data

The type of intraprocedural echocardiography, procedural time, x-ray exposure time and dose was similar between the two groups. The number of deployments and device resizing in the non-PE group were similar to those in the PE group. The average diameters of the device umbrella (31.7 mm ± 5.6 mm vs. 26.9 mm ± 5.0 mm, *P *= 0.026) and cover (36.3 mm ± 4.6 mm vs. 33.4 mm ± 4.0 mm, *P *= 0.075) were larger in the PE group than in the non-PE group. The calculated C/O ratio (1.20 ± 0.13 vs. 1.09 ± 0.08) was larger in the non-PE group, while the calculated U/l ratio was similar between the two groups (Table 2).

**Table 2:** Periprocedural data.

Variables	Non-PE	PE	*P* value
*n*	127	6	
Intraprocedural echocardiography, *n* (%)			0.423
TEE	81 (63.8)	5 (83.3)	
ICE	46 (36.2)	1 (16.7)	
Procedural time, min	63 (49, 90)	74 (45, 208)	0.488
x-ray exposure time, min	6.2 (4.0, 9.5)	8.1 (6.2, 8.1)	0.130
x-ray exposure dose, mGy	120 (53, 200)	194 (95, 289)	0.168
LAA morphology, *n* (%)			0.267
Cauliflower	81 (63.8)	3 (50.0)	
Chicken wing	17 (13.4)	1 (16.7)	
Cactus	25 (19.7)	1 (16.7)	
Windsock	4 (3.1)	1 (16.7)	
Number of deployments	1.4 ± 0.7	1.8 ± 1.0	0.164
Device resizing, *n* (%)	6 (4.7)	0	1.000
Diameter of umbrella, mm	26.9 ± 5.0	31.7 ± 5.6	0.026
Diameter of cover, mm	33.4 ± 4.0	36.3 ± 4.6	0.075
U/l ratio	1.13 ± 0.07	1.14 ± 0.06	0.878
C/O ratio	1.20 ± 0.13	1.09 ± 0.08	0.059
Complications, *n* (%)			
Stroke/TIA	0	0	1.000
Systemic embolism	0	0	1.000
Device embolization	0	0	1.000
Vascular complications	2 (1.6)	0	1.000

C/O, diameter of cover/LAA maximal orifice diameter; ICE, intracardiac echocardiography; LAA, left atrial appendage; LAAC, left atrial appendage closure; PE, Pericardial effusion; TEE, transesophageal echocardiography; TIA, transient ischemic attack; U/l, diameter of umbrella/LAA landing zone maximal diameter.

### 3.3. Management of PE

Three patients developed acute PE on the day of the LAmbre implantation. Two of the patients had taken dabigatran periprocedurally, with one patient having been in sinus rhythm (SR) and the other had been in AF rhythm. One patient took rivaroxaban and had been in AF rhythm. OAC was discontinued immediately and reinitiated before discharge. The following antithrombotic medication was prescribed as scheduled. Three individuals developed delayed PE following the procedure. PE was detected in one patient 47 days after device implantation while on warfarin and in SR. One patient developed PE 53 days following the procedure and was taking rivaroxaban and was in SR. The third patient experienced PE 155 days following device implantation and was taking both aspirin and clopidogrel and was in AF rhythm. All patients with acute and delayed PE were treated with pericardiocentesis, and none required blood transfusion (Table 3). All acute and delayed PE was hematic nature. Antithrombotic medication was switched to single antiplatelet therapy or none.

**Table 3:** Details of patients with PE.

Events	Age, years	Sex	Days after LAAC	Device size, mm	Rhythm status	Antithrombotic therapy	Treatment
Acute PE	86	Male	0	28/34	SR	Dabigatran	Pericardiocentesis
Acute PE	69	Male	0	22/28	AF	Dabigatran	Pericardiocentesis
Acute PE	67	Male	0	36/40	AF	Rivaroxaban	Pericardiocentesis
Delayed PE	66	Female	47	34/38	SR	Warfarin	Pericardiocentesis
Delayed PE	65	Male	53	36/40	SR	Rivaroxaban	Pericardiocentesis
Delayed PE	77	Male	155	34/38	AF	Dual APT	Pericardiocentesis

AF, atrial fibrillation; APT, antiplatelet therapy; LAA, left atrial appendage; LAAC, left atrial appendage closure; PE, pericardial effusion; SR, sinus rhythm.

### 3.4. Predictors of PE

Univariate logistic regression analysis revealed that congestive heart failure (OR = 6.47, 95% CI* *= 1.21–34.71, *P *= 0.029) and LAA maximal orifice diameter (OR = 1.22, 95% CI* *= 1.02–1.45, *P *= 0.027) were both correlated with PE following LAmbre device implantation (Table 4).

**Table 4:** The predictors of PE after LAmbre device implantation.

	OR	95% CI	*P* value
Age, years	1.02	0.92–1.12	0.740
Male	2.94	0.33–25.91	0.332
BMI, kg/m^2^	1.01	0.78–1.30	0.943
Nonparoxysmal AF	0.52	0.09–2.97	0.457
CHA_2_DS_2_-VASc score	1.15	0.67–1.97	0.609
HAS-BLED score	1.29	0.57–2.92	0.550
LA diameter, mm	1.02	0.91–1.14	0.712
Congestive heart failure	6.47	1.21–34.71	0.029
LVEF, %	0.92	0.83–1.02	0.099
LAA maximal orifice diameter, mm	1.22	1.02–1.45	0.027
LAA Landing zone diameter, mm	1.18	0.99–1.41	0.065
U/l ratio, per 0.1 increase	1.10	0.35–3.45	0.877
C/O ratio, per 0.1 increase	0.37	0.13–1.04	0.060

AF, atrial fibrillation; APT, antiplatelet therapy; BMI, body mass index; C/O, diameter of cover/LAA maximal orifice diameter; LA, left atrium; LAA, left atrial appendage; LAAC, left atrial appendage closure; LVEF, left ventricular ejection fraction; OAC, oral anticoagulant; PE, Pericardial effusion; U/l, diameter of umbrella/LAA landing zone maximal diameter.

### 3.5. Follow-up results

In the PE group, 1 patient underwent DRT 44 days after the procedure and totally dissolved after prolongation of OAC medication. No patients experienced stroke, systemic embolism, device embolization, or major bleeding and there were no deaths. None of the patients had recurrent PE following treatment. In the non-PE group, 1 patient died. Ischemic stroke and major bleeding occurred in 1 and 2 patients, respectively. DRT was detected in 4 patients and confirmed complete dissolution after prolonging OAC therapy. Antithrombotic medication was switched as scheduled of other patients in the non-PE group. No PDL >5 mm was detected in any patient during the follow-up TEE study.

## 4. Discussion

To the best of our knowledge, the present study was the first to investigate both acute and delayed PE following LAmbre device implantation. We found that PE was not uncommon after LAAC with the LAmbre device. Congestive heart failure and a large LAA orifice diameter were both correlated with the development of PE following LAmbre implantation.

### 4.1. Incidence of PE following LAAC

PE requiring intervention is one of the most serious complications that can occur following LAAC. The reported incidence of PE following Watchman implantation is relatively low. In a recent study from the NCDR Left Atrial Appendage Occlusion Registry, the incidence of PE requiring intervention was reported to be 1.4% (7). A real-world analysis of >17,000 recipients of the Watchman implant reported the incidence of PE requiring intervention to be 1.2% (8). The rate of PE requiring intervention following Watchman implantation was 1.1% in our previously reported study (17). In the Amulet IDE trial, the incidence of PE was higher in patients receiving the Amulet occluder (2.4% vs. 1.2%) (6). However, most of the previous studies focused on periprocedural PE, and the timing of the PE events was generally not specified. Two patients (0.2%) experienced PE within 30 days of the procedure in the EWOLUTION study (18). According to data reported to a US nationwide registry, 43 out of 13,309 subjects (0.3%) with Watchman implantation were readmitted with delayed PE/PT within 30 days (10).

There is only limited data currently reported regarding PE following LAmbre device implantation. PE occurred in 3 patients (2.0%) in a prospective multicenter clinical study of the use of the LAmbre device. Park et al. (13) reported an incidence of 3.3% (2/60) for PE in patients who underwent LAAC with the LAmbre device. The two cases were both delayed presentations (Days 8 and 33 postprocedure) and both required pericardiocentesis. Schnupp et al. (19) reported a rate of 5% (2/40) periprocedural PE requiring intervention following LAmbre device implantation. In the study of Xiao et al. (14), 4 of 224 (1.8%) patients who underwent LAmbre device implantation developed delayed PE/PT. In the present study, the incidence of acute and delayed PE was 2.5% each, which was similar to previous investigations.

### 4.2. Predictors of PE following LAAC

Most previous investigations reporting on the factors associated with the development of PE were conducted in patients who had implantation of the Watchman device implantation. Data on the predictors of PE after implantation of the LAmbre device are limited. Xiao et al. (14) reported that the only factor associated with delayed PE events was if the umbrella of the LAmbre device did not fully open. Of note, age, sex, presence of paroxysmal AF, and number of recaptures were not found to be correlated with the incidence of delayed PE. However, several other studies have demonstrated that the presence of SR at the time of implantation was correlated with PE (7, 11). It has been postulated that LAA contraction may exert force on the device, particularly at the site of anchors, leading to the development of PE. In our report, we found neither the presence of paroxysmal AF nor SR was correlated with the development of PE. The significance of these inconsistent findings across the reported studies are unclear and may be caused by the different study samples.

We found that larger LAA orifice diameter was correlated with the development of PE following LAmbre device implantation. A prior study showed that larger LAA orifice was independently correlated with PDL after Watchman device implantation (20). We postulate that the occluding devices may be relatively less stable with increasing LAA orifice size. Compared with LA myocardium, LAA myocardium demonstrates increased expansibility, and we feel that the discrepancy between the two may lead to slight movement of the device from the LAA wall, which may be more evident when the LAA opening is larger. The slight movement of the device may cause damage to the LAA wall and result in microperforation.

The presence of congestive heart failure was a predictor of occurrence of PE after LAAC in the present study. The NCDR Left Atrial Appendage Occlusion Registry also showed that left ventricular function was correlated with incidence of PE (7). Theoretically, patients with congestive heart failure may experience LA pressure overload, which may increase the risk of PE (21, 22). However, no data regarding the relationship between LA pressure and PE following LAAC have been reported. From a pathophysiology perspective, elevated LA pressure may cause alveolar-capillary network destruction and pulmonary vascular remodeling, subsequently leading to pulmonary arterial hypertension. Recently, Zou et al. (23) found that elevated pulmonary artery systolic pressure was correlated with postprocedure PE in patients with AF undergoing LAAC.

### 4.3. Potential mechanism

The majority of acute PE reported following LAAC have been bloody effusions, which suggests that they may result from injury at various points during the procedure, including transseptal puncture, manipulation of wires and sheaths, or occluders in the LA or LAA. Therefore, monitoring by the use of intraprocedural echocardiography and repeated echocardiography following the procedure are necessary to detect this complication. Previous reports have demonstrated that most delayed pericardial effusions were also bloody. The main cause of bloody delayed PE's is determined to be microperforation of the LAA caused by the occlude device. The literature suggests that patients who had nitinol plug devices (Amulet or LAmbre) implanted have a higher rate of delayed PE than those who had nitinol cage devices (Watchman) implanted. One possible explanation for this difference is that the anchor length of the nitinol plug devices are longer than that of the nitinol cage devices. Our findings may provide insight into factors associated with PE and may inform decision-making on both device selection and patient management. Comprehensive LAA evaluation should be performed to provide information to assist in device selection. Regular echocardiography follow-up examinations are recommended following LAmbre device implantation, especially in those patients with larger LAA orifices or congestive heart failure. In addition, chronic nonspecific pericardial inflammation may be another potential reason for delayed PE although this is relatively rare. We have previously reported a patient with delayed PE who responded well to administration of anti-inflammatory agents. A similar case has been reported in a patient who had undergone an atrial septal defect closure procedure. The underlying cause of this rare complication is hypothesized to be a reaction the nickel content of the device material.

### 4.4. Study limitations

Several limitations of this study should be noted. Firstly, the study was limited to a single-center's experience. Secondly, the number of patients with PE in our cohort was not sufficiently powered to conduct multivariable logistic regression. Therefore, we used a univariate model to identify predictive factors of the development of PE. Thirdly, acute and delayed PE were analyzed together due to the limited number of patients who developed PE. Further multicenter investigations with larger study samples are needed to confirm the findings of the current research.

## 5. Conclusion

In this single-center experience, acute and delayed PE were uncommon in patients with AF following the LAmbre device implantation. Congestive heart failure and larger LAA orifice were identified as the predictors of PE.

## Data Availability

The raw data supporting the conclusions of this article will be made available by the authors, without undue reservation.
